# Supplementary material for: Polyphyllin II Induces Protective Autophagy and Apoptosis via Inhibiting PI3K/AKT/mTOR and STAT3 Signaling in Colorectal Cancer Cells
Source: Int J Mol Sci. 2022 Oct 6;23(19):11890. doi: 10.3390/ijms231911890 (PMC9570434; doi:10.3390/ijms231911890)
Supplement: Supplementary file 1 [file ijms-23-11890-s001.zip › ijms-1948763-supplementary.pdf]

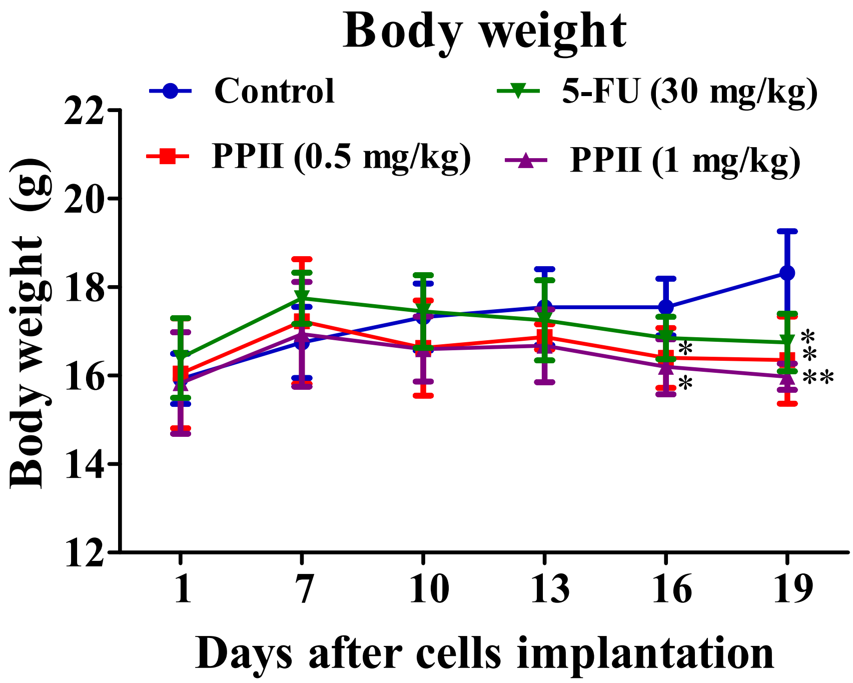


**Figure S1 Body weights of mice.** Mice were i.p. administered with vehicle, PPII (0.5 mg/kg, once every 3 days), PPII (1 mg/kg, once every 3 days) or 5-FU (30 mg/kg, positive control, once every two days) daily for 12 consecutive days. Data are presented as mean ± SD. **P*<0.05, ***P*<0.01.
